# Cellular metabolism hijacked by viruses for immunoevasion: potential antiviral targets

**DOI:** 10.3389/fimmu.2023.1228811

**Published:** 2023-07-25

**Authors:** Jiaqi Li, Yanjin Wang, Hao Deng, Su Li, Hua-Ji Qiu

**Affiliations:** State Key Laboratory for Animal Disease Control and Prevention, National African Swine Fever Para-reference Laboratory, National High Containment Facilities for Animal Diseases Control and Prevention, Harbin Veterinary Research Institute, Chinese Academy of Agricultural Sciences, Harbin, China

**Keywords:** immunometabolism, innate immunity, adaptive immunity, immunoevasion, metabolic reprogramming

## Abstract

Cellular metabolism plays a central role in the regulation of both innate and adaptive immunity. Immune cells utilize metabolic pathways to modulate the cellular differentiation or death. The intricate interplay between metabolism and immune response is critical for maintaining homeostasis and effective antiviral activities. In recent years, immunometabolism induced by viral infections has been extensively investigated, and accumulating evidence has indicated that cellular metabolism can be hijacked to facilitate viral replication. Generally, virus-induced changes in cellular metabolism lead to the reprogramming of metabolites and metabolic enzymes in different pathways (glucose, lipid, and amino acid metabolism). Metabolic reprogramming affects the function of immune cells, regulates the expression of immune molecules and determines cell fate. Therefore, it is important to explore the effector molecules with immunomodulatory properties, including metabolites, metabolic enzymes, and other immunometabolism-related molecules as the antivirals. This review summarizes the relevant advances in the field of metabolic reprogramming induced by viral infections, providing novel insights for the development of antivirals.

## 1 Introduction

Immunometabolism is involved in various disorders, including viral infections, cancers, and autoimmune diseases (1–4). Changes in the cellular microenvironment (e.g., hypoxia or pathogen invasion) result in the differences of energy requirements for cell proliferation and survival (5). Notably, viral infections usually induce the changes of cellular metabolism, which provide essential materials for the virus life cycle, including viral replication and progeny virus production (6–8). Additionally, cellular metabolic changes can alter the cellular immune responses to modulate viral replication (6).

Cellular metabolism is also indispensable for the functions of immune cells, including lymphocytes, macrophages and neutrophils. For example, glutamine is an essential nutrient for the proliferation of lymphocytes, and is also required for the immunoregulatory activities of cells (9). Interestingly, immune cells exhibit different patterns of energy metabolism in the activated and resting states (**Table 1**). Metabolic pathways are usually associated with signal transduction and the differentiation of immune cells, and different immune cell subpopulations utilize different metabolic programs depending on their state and environment. It is generally accepted that metabolic signaling determines the cell fate (21). Reportedly, inhibition of phosphoglyceraldehyde dehydrogenase (PHGDH)-mediated serine metabolism can enhance the antiviral activities of macrophages, which is due to the activation of the TBK1-IRF3 signaling pathway by the downregulation of the PHGDH-mediated ATP6V0d2 (22), indicating that PHGDH could act as a potential antiviral target in macrophages. Altogether, the interplay between viral infections and immune cell metabolism is a complex and rapidly evolving field of research.

**Table 1:** The regulation of different cellular metabolism pathways.

Cell types	Major energy metabolic pathways	Mediators	References
M1 macrophages	Glycolysis	HIF-1*α*, IRF3, and IRF5	(10)
Neutrophils	Glycolysis and glutaminolysis	HIF-1*α*	(11)
M2 macrophages	Oxidative phosphorylation and fatty acid oxidation	PPAR*γ*	(12)
Näive T cells	Oxidative phosphorylation	HIF-1*α*	(13)
Memory T cells	Oxidative phosphorylation and fatty acid oxidation	AMPK	(14–16)
Effector T cells	Oxidative phosphorylation, glycolysis, and amino acid metabolism (arginine, tryptophan, serine, leucine, glutamine, and cysteine)	c-Myc and PI3K/Akt/mTORC	(17)
Effector B cells	Oxidative phosphorylation and glycolysis	HIF-1*α*	(18)
NK cells	Glycolysis	SREBP and c-Myc	(19, 20)

Viruses depend entirely on cellular metabolism for the energy and nutrients for replication. In contrast, cellular metabolites, metabolic regulators, and metabolic enzymes involved in cellular metabolism including glucose, lipids, amino acids and nucleotide metabolism, exert antiviral activities by regulating the host immune responses (6, 23). However, these metabolic processes can be hijacked by viruses to maintain the energetic and synthetic requirements of viral progeny. Some viruses mainly activate core catabolic processes (e.g., glycolysis and the tricarboxylic acid cycle) to maintain the energy, while others mainly modulate anabolic and biosynthetic processes (e.g., nucleotide, fatty acid and amino acid synthesis) to maintain the synthetic requirements (24). More specifically, different viruses lead to multiple alterations in cellular metabolites, metabolic regulators, and metabolic enzymes in different ways, which directly or indirectly affect cellular immune responses and regulate viral infections. Previous studies have shown that infection with coronavirus (25), herpes virus (26), or African swine fever virus (27) can induce alternations in immune cell metabolism. Therefore, elucidation of the effects of viruses on cellular metabolites, metabolism-related molecules and metabolic enzymes will facilitate the development of novel antiviral strategies (28).

## 2 Interactions between glucose metabolism signaling and viral immunoevasion

Glucose is the most important energy source and the main nutrient involved in cellular metabolism (29). Cellular respiration is divided into three steps: the Embden-Meyerhof pathway (EMP), the tricarboxylic acid (TCA) cycle, and oxidative phosphorylation (OXPHOS). The intermediate product of EMP, 6-phospho-fructose, is involved in the hexosamine biosynthetic pathway (HBP), providing uridine 5’-diphosphate *N*-acetylglucosamine (UDP-GlcNAc) for target protein modifications, and is also involved in the epidermal growth factor receptor (EGFR)-mediated glucose and glutamate metabolism (30).

**Figure 1:**
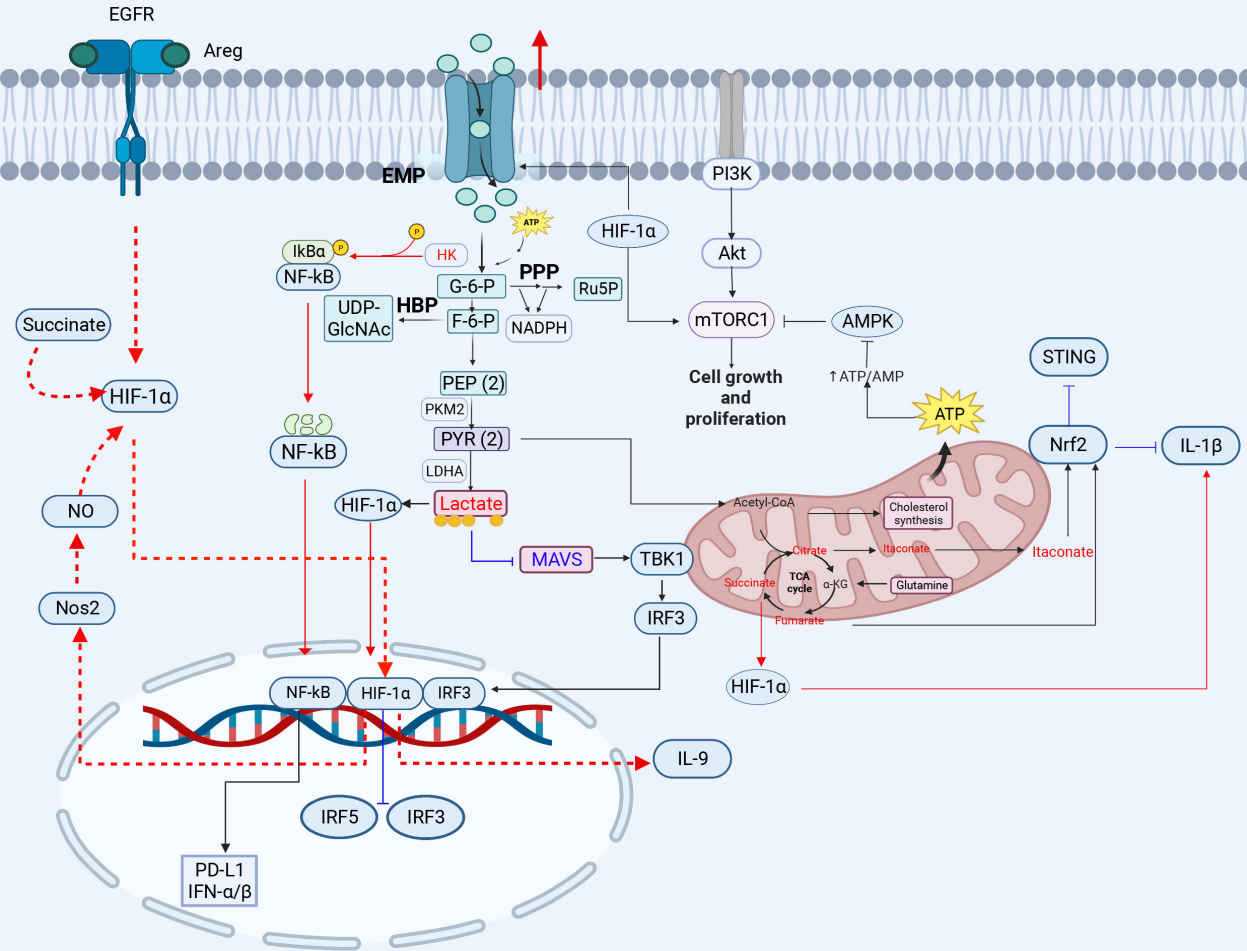
The crosstalk between glucose metabolism and innate immunity pathways. Glucose enters the cell and produces pyruvate, which is converted to lactate under hypoxic conditions. When high concentrations of fructose 6-phosphate are produced, the remaining fructose 6-phosphate enters the HBP pathway to produce UDP-GlcNAC. Acetyl-CoA enters the tricarboxylic acid cycle in the presence of sufficient oxygen, and is oxidized to produce citrate, eventually generating carbon dioxide and water, and releasing a large amount of energy. Glucose metabolism-related GLUT1, HK, LADH, OGT, HIF-1*α*, lactate, citrate, and succinate play important roles in inflammation, viral infection, and immunity (red lines indicate activation, blue lines indicate inhibition). 3-PG, 3-phosphoglycerate; 1,3-BPG, 1,3-bisphosphoglycerate; 2-PG, 2-phosphoglycerate; PEP, phosphoenolpyruvate; PYR, pyruvate; G3P, glyceraldehyde 3-phosphate; HK, hexokinase; LDH, lactate dehydrogenase; HBP, hexosamine pathway; PPP, pentose phosphate pathway. The red circle represents increased glucose intake. The figure was created by the BioRender software.

Several viruses can modulate the signaling processes involved in glucose metabolism in immune cells by hijacking cellular metabolites, metabolic regulators, and metabolic enzymes, such as glucose transporter protein (GLUT1), hexokinase 2 (HK2), and lactate (**Figure 1**). This modulation leads to a shift in energy metabolism from OXPHOS to glycolysis, which provides a substrate for the production of several biomolecules, including TCA cycle intermediates required for fatty acid anabolism and ribose phosphate pyrophosphate (PRPP) or NADPH for a base material of nucleotide anabolism to facilitate viral replication (31). Reportedly, human cytomegalovirus (HCMV) infection can promote the biosynthesis of glycolysis, metabolic enzymes, and pyrimidine nucleotides, which facilitates viral replication (32). The changes in metabolic signaling induced by viral infections require further investigation, which may guide the development of antiviral strategies.

### 2.1 The involvement of the metabolic reprogramming of enzymes in the glucose metabolism pathway and viral immunoevasion

Upregulation of glycolysis-associated GLUT and enzyme expression drives the proliferation and activation of T cells and the secretion of cytokines such as IL-2. GLUT and HK2 are vital enzymes in the glycolytic process (33). Glycolytic reprogramming involves increased expression of glucose transporter proteins, particularly GLUT1, which increases glucose uptake and allows immune cells to compete for glucose in a nutrient-restricted environment, and increased glucose uptake usually promotes viral replication (34, 35). The CD4^+^ T cells infected with human immunodeficiency virus type 1 (HIV-1) show increased glucose and glutamine metabolism due to the high-level GLUT1, which enhances HIV-1 infection (36, 37). Importantly, blockade of GLUT1 signaling or siRNA-mediated GLUT1 downregulation significantly impairs the HIV-1 infection in human T cells (38). Dengue virus (DENV) also induces the expression of GLUT1 and HK2 to promote glucose uptake and the downstream glycolytic processes to favor viral replication (39). In the rhinovirus (RV)-infected cells, viral replication is dependent on the availability of glucose and glutamine, which is affected by increasing glycogenolysis and upregulating GLUT1 expression (17). The envelope protein of white spot syndrome virus (WSSV) hijacks GLUT1 to promote viral infection (40). Furthermore, the Epstein-Barr virus (EBV) LMP1 protein can enhance transcription of GLUT1, which promotes aerobic glycolysis and the tumorigenic growth of NPC cells through the mTORC1/NF-κB signaling (41). The elucidation of interplay between GLUT1 and viruses will provide a direction for the development of effective antiviral strategies.

HK2 is an important enzyme in the glycolytic pathway and a kinase that modifies the phosphorylation of proteins to regulate immune signaling pathways. HK2 can activate the NF-*κ*B signaling pathway by phosphorylating and degrading I*κ*B. It has been demonstrated that aberrantly activated aerobic glycolysis in tumor cells promotes the segregation and binding of HK2 from mitochondria to cytoplasmic I*κ*B, where HK2 acts as a protein kinase to phosphorylate I*κ*B, leading to I*κ*B degradation and NF-*κ*B activation-dependent PD-L1 expression to escape tumor immunity (42) (**Figure 1**). Notably, the NF-*κ*B p65 plays a key role in the HBx-induced spontaneous hepatocellular carcinoma. The hepatitis B virus (HBV) x protein can activate the NF-*κ*B signaling via the p65 phosphorylation by HK2 to promote immunoevasion of the virus and enhance glycolysis, further activating PI3K/Akt signaling to increase hepatocyte proliferation (43). HK2 and lactate can suppress the activation of the retinoic acid-inducible gene I (RIG-I)-mitochondrial antiviral signaling protein (MAVS) pathway by inhibiting MAVS. HK2 and glycolysis-derived lactate also play important roles in the immunoevasion of HBV and the regulation of energy metabolism in innate immunity during HBV infection. A previous study showed that HBV isolates MAVS from RIG-I by forming a ternary complex including hexokinase (HK), which inhibits the RLR signaling pathway through LDHA-dependent lactate production (44). Influenza A virus (H1N1) utilizes HK2 and the pyruvate kinase M2 (PKM2) to enhance glycolysis and further promote viral replication (45). These studies highlight the importance of the HK enzyme in regulating viral infection through the regulation of innate immune signals, revealing the possibility of a potential antiviral target.

Lactate dehydrogenase (LDH) is a crucial enzyme in glycolysis in inflammatory macrophages (M1 phenotype). Lactate dehydrogenase B (LDHB) can regulate the NF-*κ*B signaling pathway through the mitochondrial autophagy pathway to facilitate viral replication. LDHB negatively regulates classical swine fever virus (CSFV) growth, whereas CSFV infection inhibits LDHB production and reconstructs the glycolytic metabolic pathway in immune cells by the NS3 protein (46, 47). Interestingly, LDHA expression restricts viral protein synthesis in the ARV-infected cells, whereas the avian reovirus (ARV) *σ*A protein inhibits LDHA expression and upregulates HIF-1*α* and glycolytic enzymes to promote glycolysis level, thereby favoring viral replication (48). Furthermore, LDH can increase interferon gamma (IFN-*γ*) expression by promoting histone acetylation (49). Reportedly, glucose consumption is restricted by the inhibition of LDH in CD4^+^ T cells, thus reducing the production of acetyl-CoA, which leads to insufficient histone acetylation modification and reduced IFN-*γ* production (49). However, the effect of LDH on histone acetylation during virus replication remains largely unknown, and requires further investigation.

Hexosamine, a key metabolite linking glucose metabolism and immunity, activates inflammasomes, innate immune signaling pathways and autophagy. *N*-acetylglucosamine transferase (OGT) is a critical enzyme in the hexosamine pathway that mediates the glycosylation of target proteins with UDP-GlcNAc to regulate host antiviral immunity and influence viral infections. Hexosamine can induce activation of the inflammatory response and inflammatory cytokine expression through crosstalk between the hexosamine biosynthetic pathway and *O*-GlcNAc protein modification (50). OGT overexpression promotes *O*-GlcNAc modification of thioredoxin interacting protein (TxNIP) to facilitate the interaction between TxNIP and NLRP3, which promotes IL-1*β* production in HEK293T or INS1 832/13 cells (51). OGT is important for NLRP3 activation; however, its activation during viral infections remain largely unknown and requires further investigation. Alternatively, innate immunity can be activated by *O*-GlcNAc protein modification to regulate viral infection, such as influenza A virus (IAV) and Vesicular stomatitis virus (VSV) (52). A previous study showed that supplementation with D-glucosamine increases *O*-GlcNAc modification of MAVS to inhibit viral replication, which protects mice against IAV or vesicular stomatitis virus (VSV) challenge (52). During IAV infection, OGT mediates the *O*-GlcNAcylation of IFN regulatory factor 5 (IRF5) on serine-430, and IAV utilizes the hexosamine pathway to increase the expression of proinflammatory cytokines by stimulating IRF5 leading to in tissue injury (50). VSV infection can enhance HBP activity and *O*-GlcNAcylation of the downstream protein MAVS at S366, which leads to ubiquitination of the K63 link of MAVS to activate downstream antiviral signals in macrophages (53). Hexosamine can also activate the autophagy pathway to regulate viral infections, a catabolic process that is essential for maintaining cellular homeostasis. It has been shown that low-levels glucose cause decreases in UDP-GlcNAc levels and induce autophagy via the AMPK-Akt/mTOR signaling in the HBV-infected cells (54). Low-level hexosamine can promote HBV replication by inducing autophagosome formation and inhibiting autophagic degradation *in vitro* and *in vivo* (54, 55). In addition, low-dose glucosamine treatment promotes the replication of IAV, enterovirus 71 (EV 71), and VSV *in vitro* (55). *O*-GlcNAc modification can regulate iron death through iron autophagy and mitochondrial autophagy (56). SNAP-29 is one of the components of the soluble *N*-ethylmaleimide-sensitive factor attachment protein receptor (SNARE) complex that promotes autophagic vesicle maturation. A recent study revealed that OGT-mediated SNAP-29 *O*-GlcNAc modification in nematode-infected mammalian cells facilities the activation of autophagy, suggesting that OGT could indirectly promote the maturation of autophagic vesicles in cells (57) (**Table 2**).

**Table 2:** The relationship between OGT and immunometabolism.

Pathways involving OGT	Regulated proteins	Mechanisms		Responses to *O*-GlcNAcylation	Pathogens	References
Innate immune signaling pathways	IRF5	OGT mediates the *O*-GlcNAcylation of IRF5 on serine 430 to promote IRF5 activation		Promoting anti-viral response	IAV	(50, 52)
	MAVS	OGT-mediated *O*-GlcNAcylation of MAVS on S366 promotes its K63-linked ubiquitination to activate antiviral signals		Promoting anti-viral response	VSV	(52, 53)
Inflammatory response	NLRP3, TxNIP	TxNIP *O*-GlcNAcylation in INS1832/13 cells is modulated by OGT or OGA activity to activate NLRP3		Promoting IL-1*β* production	Unknown	(51)
Autophagy	SNAP-29	OGT-mediated *O*-GlcNAcylation of SNAP-29 is on Ser 2, Ser 61, Thr 130, and Ser 153		Autophagy inhibition	*C. elegans*	(57)
	Akt and mTOR	Akt and mTOR undergo *O*-GlcNAcylation		antagonizing autophagosome-lysosome fusion and inhibiting viral replication	HBV	(55)

### 2.2 The interplay of activation of metabolism-related molecules in the glucose metabolic pathway and viral immunoevasion

HIF-1*α* regulates the gene transcription of key enzymes (GLUT1, PKM2, LDHA etc.) (**Figure 1**) in the glycolytic pathway, which is involved in the modulation of immune responses. Due to its major role in the regulation of glycolysis, HIF-1*α* can be targeted and hijacked by viruses for viral infections. A recent study has shown that Newcastle disease virus (NDV) infection results in the inhibition of TCA cycle flux and an increase in EMP flux through stable expression of HIF-1*α*, indicating a shift in energy metabolism from OXPHOS to EMP, which promotes the viral replication, indicating that NDV infection induces mitochondrial damage and hijacks cellular metabolism to promote a shift toward glycolysis to favor viral replication (58). Additionally, under hypoxic conditions, the Kaposi’s sarcoma-associated herpesvirus (KSHV) vGPCR protein induces stable expression of the HIF-1*α* protein, which leads to metabolic Warburg phenotypic changes associated with KSHV and promotes viral replication in the KSHV-infected PBMCs (18). SARS-CoV-2 infection triggers mitochondrial reactive oxygen species (ROS) production, which stabilizes HIF-1*α* and promotes glycolysis, thereby contributing to SARS-CoV-2 infection and cytokine production in SARS-CoV-2-infected monocytes and macrophages (59, 60). Therefore, this broad-spectrum effect of HIF-1*α* on viruses reveals its importance. Moreover, HIF-1*α* regulates Th9 cell differentiation and exerts antitumor functions. TGF-*β*1 and IL-4 promote naive CD4^+^ T cells to differentiate into immunoregulatory IL-9-producing helper T (Th9) cells and induce EGFR expression in the EGFR-HIF-1*α* pathway. EGFR is activated upon binding to the ligand double-regulated protein (AREG), which triggers downstream signaling via HIF-1*α* and activates the IL-9 and NOS2 promoters, facilitating IL-9 production (61). Notably, HIF-1*α* is a transcriptional repressor of IRF5 and IRF3 (62), which inhibits the production of type I IFNs. For example, SARS-CoV-2 infection triggers an inflammatory cascade, leading to elevated HMGB1 levels that inhibit IRF5-mediated type I IFN production under hypoxic conditions, only through the NF-*κ*B signaling pathway to trigger monocytes to produce inflammatory cytokines. High mobility group box 1 (HMGB1) can activate NF-*κ*B, IRF3 and IRF5 to release proinflammatory cytokines and I-IFN under normoxia, but SARS-CoV-2 infection can enhance HIF-1*α* expression, which inhibits IRF3 and IRF5 activity, leading to severe disease in COVID-19 patients (62). HIF-1*α* is a key activator of inflammatory responses in the SARS-CoV-2-infected PBMCs. HIF-1*α* expression is correlated with immune-inflammatory cytokine production. SARS-CoV-2-encoded ORF3a induces mitochondrial damage and mitochondrial-ROS production, thus promoting the stable expression of HIF-1*α* and resulting in cytokine production to enhance the inflammatory response, which is also observed in the VSV or SV-1 infection (58). In particular, HIF-1*α* is regulated by glycolytic metabolites and further stabilized by succinate and nitric oxide (NO) (61). *α*-Ketoglutarate (*α*-KG), a TCA cycle metabolite, is regulated by HIF-1*α* in Th9 cells. *α*-KG negatively regulates the stability of HIF-1*α*. Succinate increases the stability of HIF-1*α* by impairing prolyl hydroxylase 2 (PHD2) activity, thereby enhancing the antitumor activities of Th9 cells and macrophages (61, 63). HIF-1*α* can also promote programmed death ligand 1 (PD-L1) expression (64) to inhibit T-cell activation and promote immunoevasion in tumor cells. The high-risk human papilloma virus (HR-HPV) E1/E6 increases HIF-1*α* levels in cervical cell lines, thereby enhancing the Warburg effect (65–67).

### 2.3 The crosstalk of the metabolic reprogramming of metabolites in the glucose metabolic pathway and viral immunoevasion

Lactate is an end product of the glycolytic pathway. Lactate can promote the conversion of macrophages to M2 macrophages by modifying histones and regulating polarization-related genes (68), lactate promotes the formation of immunosuppressive M2 macrophages via the induction of arginase differentiation after lactate uptake by macrophages. In addition to modifying histones, lactate regulates viral immunoevasion through innate immune signaling pathways. MAVS is the protein downstream of RIG-I and is involved in crosstalk between the antiviral immune signaling pathways and glycolytic metabolic pathways. Basically, MAVS links energy metabolism and innate immunity through the recognition of lactate (69). Inactivation of glycolysis is essential for the promotion of RLR-mediated type I IFN production. Upon activation of RLR signaling, activated RIG-I “traps” MAVS through the CARD domain to activate downstream molecules and produce IFNs. Then, the oligomerized MAVS dissociates from HK2, which results in a reduction of HK activity, which in turn leads to the inhibition of glycolysis (33). Remarkably, viral infections promote glycolysis, and excess lactate inhibits MAVS and type I IFN production by binding to the transmembrane (TM) domain of MAVS directly and preventing its aggregation. The increased lactate levels in the ASFV-infected porcine alveolar macrophages (PAMs) can inhibit IFN-*β* production via the RIG-I-MAVS signaling pathway, which can contribute to viral immunoevasion (70). HBV inhibits RLR signaling via the LDHA-dependent lactate production (44). Similarly, LDHA-dependent lactate promotes IAV replication by inhibiting the MAVS-dependent type I IFN response (71). However, LDHA-dependent lactate inhibits SARS-CoV-2 replication (71). There exists interplay between lactate and innate immune responses, indicating that lactate could act as an antiviral target.

Itaconate, a derivative of the TCA cycle citrate, exhibits anti-inflammatory effects and is closely associated with the cGAS-STING signaling pathway (9). A recent study showed that the cell-permeable derivative of itaconate (4-octyl-itaconate, 4-OI) inhibits IL-1*β* transcription by activating the transcription factor Nrf2, which acts as a transcriptional repressor of IL-1*β* (72). Inhibition of STING expression is observed during metabolic reprogramming of TLR signaling, and it can also be induced by the TCA cycle via the activation of Nrf2. 4-OI, a cell-permeable derivative of the metabolite oxalic acid, can activate Nrf2 to induce an IFN-independent antiviral program, thereby inhibiting viral replication and suppressing inflammatory responses induced by SARS-CoV-2 infection (72). Furthermore, it has been reported that treatment with Nrf2-inducing agents, such as 4-OI, can sufficiently reduce the STING-dependent type I IFN release in the SAVI-derived fibroblasts, reducing the STING-associated inflammatory disease (9).

Succinate is a TCA cycle intermediate that contributes to the inflammatory response or apoptosis through stabilization of HIF-1*α* expression or succinylation of proteins (73). It has been shown that succinate can stabilize HIF-1*α* expression and then induce the expression of IL-1*β* (**Figure 1**). Succinate is an endogenous danger signal that stabilizes HIF-1*α*, which specifically regulates the expression of IL-1*β* and other HIF-1*α*-dependent genes, leading to protein succinylation and playing an important role in cell apoptosis or tumor proliferation (63, 74). Succinate promotes HIF-1*α* stabilization by inhibiting PHD2 activity in macrophages. Succinate can induce the expression of IL-9, HIF-1*α* and Th9-related genes in Th9 cells, and the induction of Th9 cell differentiation by succinate may occur via inhibiting PHD2 and promoting HIF-1*α* activity; therefore, succinate treatment enhances the antitumor activity of Th9 cells (61). Succinate is oxidized by succinate dehydrogenase to produce fumarate. Fumarate and its derivatives monomethyl fumarate (MMF) and dimethyl fumarate (DMF) are potent immunomodulators and antioxidants that can activate Nrf2 and modulate oxidative stress in cells, which can suppress HIV replication (75). The Nrf2 agonist DMF significantly inhibits SARS-CoV-2 replication and the expression of inflammatory genes (72). This inhibitory effect has also been observed in infection with viruses, such as herpes simplex virus 1 and 2 (HSV-1 and -2), vaccinia virus (VACV), or Zika virus (ZIKV), indicating that Nrf2 exhibits the extensive antiviral activities (72).

## 3 Interactions between lipid metabolism signaling and viral immunoevasion

Lipid is the general term for fats and lipids, such as triacylglycerols or triglycerides, but lipids also include phospholipids, glycolipids, cholesterol and its esters.

**Figure 2:**
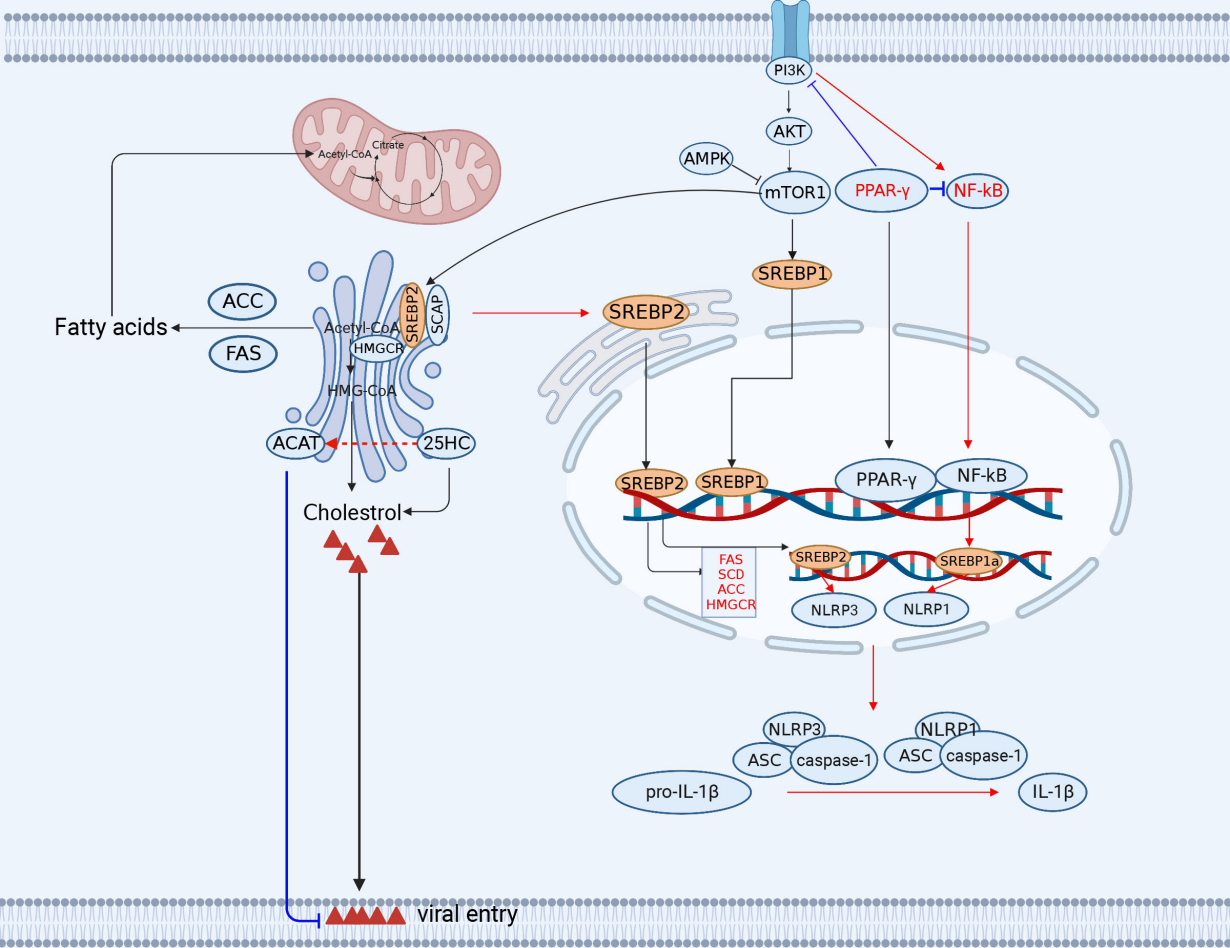
The engagement of fatty acid and cholesterol metabolism in innate immunity. The TCA cycle provides the precursor (citrate) for fatty acid synthesis (FAS). Acetyl-CoA synthesizes a variety of acetyl-CoA-based fatty acids *de novo* through the further activity of ACC and FASN using fatty acids. Lipid metabolism-related SREBP, 25HC, FANS, SCD2/ACC, and PPAR-*γ* play important roles in antiviral immunity (red lines indicate activation, blue lines indicate inhibition). ACC, (acetyl-CoA carboxylase); FANS, (fatty acid synthase); SREBP, sterol regulatory element binding protein; CH25H, cholesterol 25-hydroxylase. The figure was created by the BioRender software.

Fatty acid biosynthesis provides sufficient substrates for viral replication. There are two key metabolic enzymes involved in fatty acid biosynthesis, acetyl-CoA carboxylase (ACC) and fatty acid synthase (FASN). Notably, the expression of both FASN and ACC, is regulated by the sterol regulatory element binding protein 1 (SREBP1) family members 1a and 1c, which can regulate fatty acid synthesis (76, 77) (**Figure 2**).

Cholesterol is mainly localized in the cell membrane and also involved in the immunoevasion of viruses. In metabolically reprogrammed cells, cholesterol is thought to be a substance with several different biological functions, such as immunosuppression, viral replication (78), attachment (79), and entry (80).

The metabolism of cholesterol and fatty acids can be reprogrammed during viral infections, which regulates the host immune responses as well as viral replication. Therefore, understanding the potential mechanisms underlying metabolic reprogramming of cellular metabolites, metabolic regulators, and metabolic enzymes in the cholesterol and fatty acid metabolic pathways during viral infections can provide new insights into the development of therapeutic strategies for combating viral infections, such as ACC and SREBP.

### 3.1 The association of the fatty acid metabolic pathway and viral immunoevasion

The enzymes associated with the fatty acid biosynthesis pathway not only meet the requirements of the viral life cycle but also participate in the antiviral response. Reportedly, deletion of the fatty acid synthesis-related genes *SCD2* and *ACC* results in reduced production of monounsaturated fatty acids (MUFAs), leading to the activation of the cGAS-STING pathway-mediated interferon-stimulated gene (ISG) expression due to the spontaneous production of IFN-*α* by CD4^+^ T cells, thereby increasing the antiviral activities of IAV (81). Furthermore, a negative feedback regulatory pathway between STING and FADS2/SCD1 fine-tunes the generation of polyunsaturated fatty acids (PUFAs) associated with inflammatory response, which in turn suppresses the STING-related inflammatory response (82).

The members of the SREBP family modulate the T-cell metabolism and the gene transcription of immune-associated molecules. CD8^+^ T cells with deficient SREBP activity are unable to undergo metabolic reprogramming and blastogenesis, which results in the inability to generate a functional T-cell response (83). Furthermore, the SREBP1c isoform is involved in Th17 cell differentiation and binds directly to the IL-17 promoter to suppress the AhR-induced IL-17 expression in CD4^+^ T cells (84). In addition, the SREBP1a isoform is required for myeloid cells to exert proinflammatory effects, including the secretion of IL-1*β*, which promotes the expression of NLRP1, a key component of the inflammasome (85). Notably, SREBP1a is required for macrophages to exert proinflammatory effects, including secretion of IL-1*β* and the expression of NLRP1 (a key component of the inflammasome) (85) (**Figure 2**).

NF-*κ*B inhibition induced by the peroxisome proliferator-activated receptor gamma (PPAR-*γ*) activation is the major reason for virus hijacking of fatty acid metabolism to trigger immunoevasion (86). PPAR-*γ* activation inhibits HIV genomic LTR promoter activity and suppresses the NF-*κ*B response, thereby reducing NF-*κ*B occupancy of the LTR promoter in the infected cells and ultimately impairing HIV replication in macrophages (87). Although both the HIV and HCMV genomes contain elements responsive to NF-*κ*B, the major immediate early promoter (MIEP) contains two PPAR response elements (PPRE) in the HCMV-infected cells. In fact, PPAR-*γ* promotes viral replication through transactivation of the HCMV promoter MIEP by two PPREs in the HCMV-infected cells (88). The increased activity of PPAR-*γ* may promote both viral replication and host cell survival. Overexpression of PPAR-*γ* promotes IAV replication by inhibiting IFN signaling in alveolar macrophages (89).

### 3.2 The correlation of the cholesterol metabolic pathway and viral immunoevasion

Apolipoprotein E (ApoE) is linked to a variety of immune responses, including the suppression of T-cell proliferation, the modulation of macrophage function, lipid antigen transport to natural killer T cells, and the regulation of inflammation or oxidation. Proinflammatory cytokines induce the downregulation of ApoE in monocytes. However, TGF-*β* promotes the expression of ApoE. In addition, ApoE is required for lipoprotein transport. ApoE is known to promote the entry, assembly, and transmission of hepatitis C virus (HCV) (90–92). ApoE has been shown to modulate IAV infectio*n in vitro* and *in vivo*, and ApoE knockout markedly increases the susceptibility of mice to IAV. Notably, when cells are unable to synthesize ApoE, cell cholesterol homeostasis is disrupted to promote IAV attachment (93).

Cholesterol 25-hydroxylase (CH25H) is the key enzyme that regulates cholesterol metabolism and catalyzes the conversion of cholesterol to a soluble, broad-spectrum antiviral effector (25-hydroxycholesterol (25HC)) by adding a second hydroxyl group at position 25 (94). Interestingly, CH25H as an antiviral ISG, forms a part of the sterol metabolic network through interferon signaling (95, 96). It has been shown that CH25H activity is induced by the STAT1-dependent proinflammatory factor IL-1*β*/TNF-*α*/IL-6 in the ZIKV-infected cells (96). In addition, 25HC can inhibit virus-induced intercellular membrane fusion and viral infections. ZIKV infection can markedly increase the expression levels of CH25H in cells, thus augmenting the production of 25HC to prevent the virus entry by blocking virus-mediated membrane fusion between cells (97). Interestingly, 25HC can activate an acyl coenzyme A-cholesterol acyltransferase (ACAT) in the endoplasmic reticulum (ER) of cells, which subsequently eliminates accessible cholesterol from the cell membrane, thereby inhibiting viral entry. SARS-CoV-2 initially binds to human lung cells via the ACE2 receptor, and cholesterol in the cellular membrane is required for the membrane fusion of the virus (94). CSFV infection same as well (98) Therefore, 25HC can inhibit the virus entry. Notably, 25HC and 27HC not only inhibit viral entry but also induce the production of proinflammatory cytokines and suppress viral immune evasion. It has been demonstrated that the stimulation of oxysterols enhances HSV-1-induced IL-6 production, indicating that IL-6 exerts antiviral effects during HSV-1 infection, which is an additional antiviral mechanism of action for 25HC and 27HC (99).

Cholesterol is involved in the immunoevasion of viruses. High-levels cholesterol can weaken the host’s antiviral immunity. The PRRSV Nsp4 protein upregulates protein phosphatase 2 (PP2) activity, which activates the rate-limiting enzyme 3-hydroxy-3-methylglutaryl coenzyme A reductase HMGCR in the cholesterol synthesis pathway and increases cellular cholesterol levels, thereby inhibiting virus-induced IFN-*β* production and promoting PRRSV replication (100). CSFV infection modulates the cholesterol biosynthesis pathway to favor the virus entry and disrupt the type I IFN response (101, 102). On the contrary, low-levels cholesterol may enhance antiviral immunity in host cells. However, a reduction in cholesterol biosynthesis also enhances host antiviral immunity. When cholesterol synthesis is decreased, STING, a critical antiviral signaling protein, is stimulated to generate antiviral responses. STING appears to require a cholesterol-deficient ER membrane to promote type I IFN production (103) (**Figure 2**). In summary, CH25H is a molecule with broad spectrum antiviral activity.

SREBP2 is involved in cholesterol metabolism. The maturation of SREBP2, a master transcription factor for cholesterol metabolism, regulates NLRP3 inflammasome activation through the translocation of the SCAP-SREBP2 complex from the ER into the Golgi, thus promoting the activation of the NLRP3 inflammasome. NLRP3 has been reported to form ternary complexes with the SREBP cleavage-activating proteins SCAP and SREBP2, which in turn translocate proximally to the Golgi apparatus to form mitochondrial clusters for efficient assembly of the inflammasome (104).

## 4 Interactions between amino acid metabolism signaling and viral immunoevasion

Amino acid metabolism also known as one carbon metabolism, plays important roles in viral replication and immune regulation (105).

**Figure 3:**
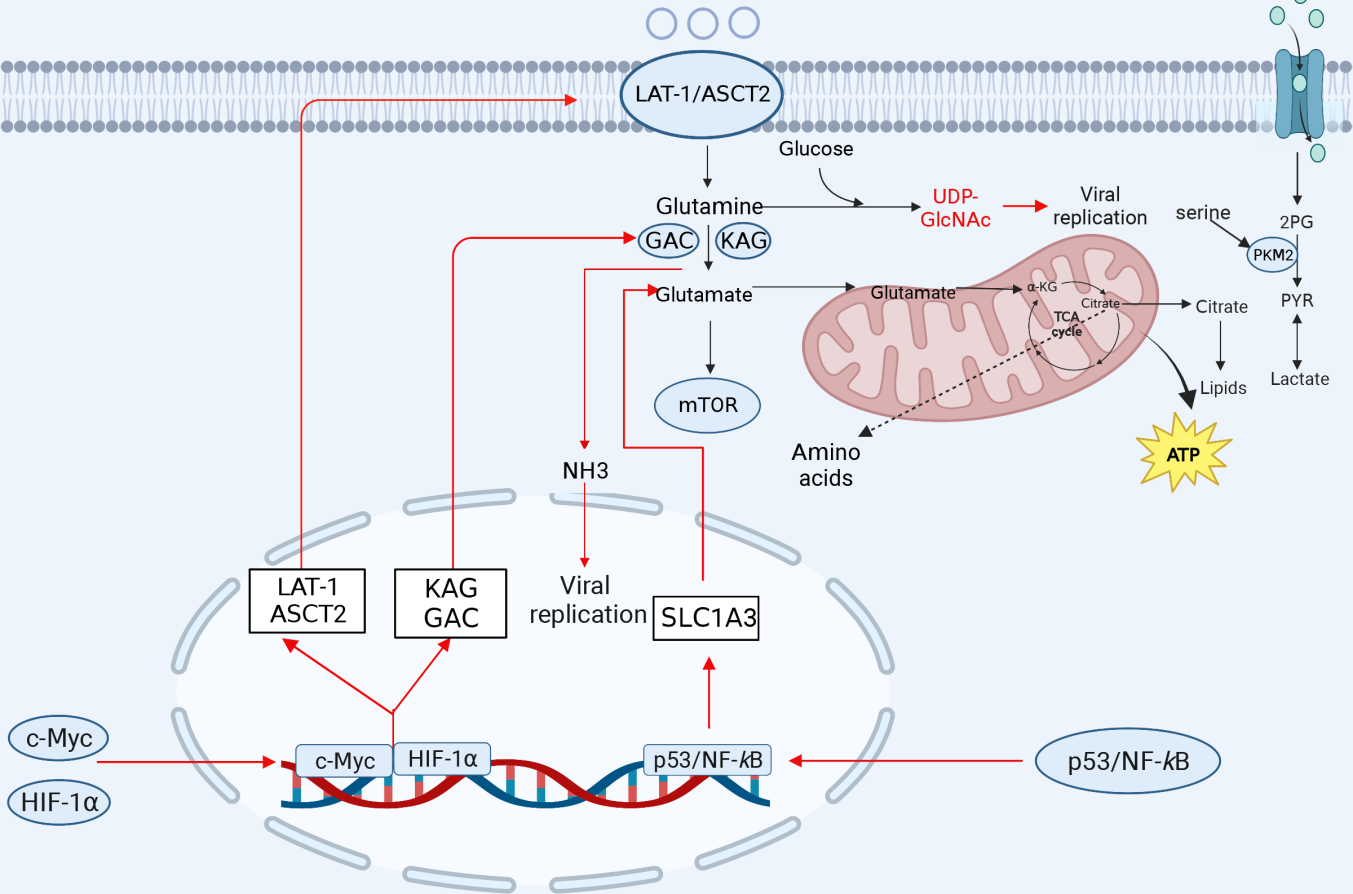
The involvement of amino acid metabolism in innate immunity. Glutamine is transformed into glutamate after entering the cell to facilitate the TCA cycle repair. Glutamate is transformed to *α*-KG, which is integrated into the TCA cycle and oxidized to generate ATP for energy. Glutamine can produce a variety of amino acids. Serine is linked to several glycolytic enzymes. Serine aids aerobic glycolysis and lactate production by activating PKM2. Amino acid metabolism-related ASCT2, citrate, NH3, glutamine, and *α*-KG play important roles in viral replication. After viral infection, some transcription factors can induce a reprogramming of glutamine metabolism, leading to increased expression of the genes involved in glutamine uptake and metabolism, which promotes viral replication (red lines indicate activation, blue lines indicate inhibition). KGA, kidney-type glutaminase; GAC, glutaminase C. The figure was created by the BioRender software.

Generally, viral infections can affect the host’s immune response by inducing changes in host cell glutamine metabolism and tryptophan metabolism. Therefore, metabolic reprogramming of glutamine and tryptophan plays a crucial role in viral immunoevasion, and exploring the mechanisms will help provide an in-depth understanding of viral infections and immunoevasion (**Figure 3**).

### 4.1 Metabolic reprogramming of glutamine regulates the viral immunoevasion

Glutamine is critical for viral replication, and viral infection regulates the metabolism of glutamine depending on glucose levels. Several viruses transfer carbon to biosynthetic reactions via aerobic glycolysis, using glutamine to replenish intermediates of the TCA cycle, and then reprogramming cellular metabolism to provide the energy required for viral replication and the molecular material for the production of progeny virus. For example, in the VACV-infected HeLa or 2FTGH cells, HIF-1*α* mediates the shift from glucose to glutamine metabolism via the viral C16 protein, and stimulation of the viral protein contributes to glutamine metabolism to produce *α*-ketoglutarate and some of the macromolecular precursors of TCA cycle metabolism, which is dependent on glutamine, and asparagine, in the absence of glutamine to promote the production of VACV proteins (39, 106). In the HIV-infected CD4^+^ T cells, the carbon generated from glutamine catabolism enters the TCA cycle to facilitate HIV infection, which is also necessary for maintaining the balance between the TCA cycle and oxidative phosphorylation (107). It has also been shown that glutaminase: kidney-type glutaminase (KGA), glutaminase C (GAC), phosphoribosyl pyrophosphate aminotransferase (PPAT) (key enzymes for *de novo* purine nucleotide synthesis) and glutamine-fructose-6-phosphate transaminase 1 (a key enzyme in the hexosamine pathway) are significantly differentially expressed after glucose deprivation in the HIV-infected CD4^+^ T cells (35), indicating that the utilization of the glutamine metabolic pathway significantly promotes HIV replication. Glutamine is essential for RGNNV replication as it is converted to *α*-KG by the enzyme glutaminase (GLS), which is involved in the TCA cycle, and the process promotes RGNNV replication through the TCA cycle (108). HCMV infection increases the glutamine catabolism, which produces *α*-KG to replenish the TCA cycle and promote HCMV replication (109). In addition, ASFV infection dramatically induces the activation of intracellular glutamine metabolism and facilitates the viral replication in alveolar mononuclear macrophages (27). Furthermore, EV 71 infection can be promoted by increasing glutamine catabolism, while pharmacological inhibition of pyrimidine metabolizing enzymes can inhibit viral replication in Vero cells (110).

Glutamine produces glutamate and glutathione, an antioxidant compound that plays an important role in the prevention of oxidative stress. Similarly, HCV infection increases the utilization and dependence on glutamine, whereas inhibition of glutamine metabolism attenuates the viral infection and the cellular oxidative stress (111). The expression of Myc, two transporter proteins (SLC1A5 and SLC7A5) and two GLSs (KGA and GAC) was increased in the HCV-infected Huh7.5 cells (**Figure 3**). Myc is a transcription factor that induces the transcription of transporter proteins, thereby driving increased cellular glutamine utilization and thus metabolic reprogramming of cells. Inhibition of glutamine metabolism reduces viral replication and oxidative stress induced by HCV infection, suggesting that the inhibition of glutamine utilization is a novel strategy for the treatment or prevention of viral infection (111).

Glutamine metabolism plays a crucial role in regulating antiviral immunity, as evidenced by the inhibition of the viral infections through glutamine degradation. For example, the proliferation of HSV-infected T cells requires the metabolic breakdown of glutamine. Interestingly, glutamine supplementation has been shown to inhibit the reactivation of latent HSV through a mechanism involving enhancement of the IFN-*γ*-related immune response, which in turn inhibits the activation of the latent virus (112).

SLC1A3 (aspartic acid transporter, GLAST) is a member of the solute family and is significantly upregulated through the P53 and NF-*κ*B pathways in the NDV-infected DF-1 or A549 cells, and SLC1A3 hijacked by NDV promotes glutamate catabolism and viral replication in host cells (113).

Glutamine has been shown to produce glucosamine, which in turn allows the modification of key proteins through acetylation. This modification helps to regulate cytokine production downstream, which can affect the antiviral immune response in host cells. The process of producing glucosamine involves the catalysis of glutamine and fructose-6-phosphate by glutamine fructose-6-phosphate amidotransferase (GFAT), generating glucosamine-6-phosphate. Finally, UDP-GlcNAc induces modification of the *O*-GlcNAc protein.

### 4.2 Tryptophan metabolism and viral immunoevasion

The kynurenine pathway is a complex network of enzymatic reactions that metabolizes tryptophan into various downstream metabolites, including kynurenine, quinolinic acid, and picolinic acid. These metabolites are involved in the modulation of immune responses, oxidative stress, and neurotransmitter synthesis. Tryptophan degradation is primarily mediated by the conversion of tryptophan to kynurenine by two different dioxygenases: indoleamine-2,3-dioxygenase (IDO1) and tryptophan-2,3-dioxygenase (TDO2); the rate-limiting step in the tryptophan-kynurenine pathway is performed by IDO1. IDO1 is a multifunctional enzyme that typically acts as a negative regulator of inflammatory and immune responses (114, 115). IDO is a critical regulator of acute pulmonary inflammation. IDO deficiency severely exacerbates lung inflammation in mice. Therefore, IFN signaling in the lung parenchyma inhibits idiopathic pneumonia syndrome by promoting IDO expression (116). It has been demonstrated that changes in tryptophan metabolism are associated with IL-6 levels. In the context of viral infection, IFNs can enhance tryptophan metabolism by inducing IDO1 production subsequently leading to alterations in metabolism and inflammation, which ultimately affect viral replication (117, 118). SARS-CoV-2 infection induces metabolic reprogramming of tryptophan toward the kynurenine pathway, which regulates host inflammation and immunity (119). Furthermore, IFN-*γ*-induced antiviral activity against measles virus can be counteracted by the addition of excess tryptophan (120). In the context of SARS-CoV-2 infection, dysregulation of the kynurenine pathway has been associated with severe disease outcomes, including cytokine storm and respiratory failure.

## 5 Other potential targets of immunometabolism

Methylenetetrahydrofolate dehydrogenase 2 (MTHFD2) reductase is an important enzyme in folate metabolism. Folate plays an indirect or direct role in cell proliferation and differentiation. Reportedly, EBV infection remodels B-cell metabolism and hijacks serine metabolism by upregulating MTHFD2 to promote rapid B-cell growth, which leads to B-cell lymphoma (121). MTHFD2 is a metabolic checkpoint controlling effector and regulatory T-cell fate and function. The MTHFD2 regulates purine synthesis and signal transduction in activated T-cells to promote the cell proliferation and inflammatory cytokine production (122). NDV hijacks MTHFD2 of the nucleotide pathway to maintain nucleotide availability required for viral replication, revealing the dependence of NDV on the cellular oxidative pentose phosphate pathway (PPP) and folate-mediated one-carbon metabolism (123). Thus, MTHFD2 is a metabolic checkpoint, which is combined purine metabolism with the pathogenic effector cell signaling pathway, indicating that MTHFD2 is a potential therapeutic target in the one-carbon metabolic pathway.

Isocitrate dehydrogenase (IDH) is a key rate-limiting enzyme in the TCA cycle and plays an important role in energy metabolism, which is a noteworthy potential therapeutic target. On one hand, IDH mutation can generate high levels of 2-hydroxyglutaric acid (2-HG), which inhibits glioma stem cell differentiation (124). On the other hand, IDH mutation can also induce high levels of HIF-1*α* to promote glioma invasion (125). Reportedly, the ARV σA protein can promote viral replication by upregulating IDH3B and glutamate dehydrogenase (GDH) to promote glutaminolysis (48). Importantly, IDH has been clinically useful as a therapeutic target for acute myeloid leukemia (126).

## 6 The metabolic enzymes as potential antiviral targets

Metabolic reprogramming is an important characteristic of viral infections. To date, abnormal glucose metabolism has been extensively studied. In the presence of sufficient oxygen, glycolysis is the principal glucose metabolic pathway in several virus-infected cells. In addition, metabolic enzymes are attractive potential therapeutic targets. To date, few drugs targeting metabolism are available. We summarize recent progress in the virus-induced metabolic changes and the drugs (**Table 3**) to provide novel strategies for targeting metabolism to inhibit viral replication *in vivo* or *in vitro*. Generally, the identified antiviral targets are associated with the enzymes in cellular metabolism, it is not clear whether targeted metabolic enzymes will induce cytotoxicity, thus the future clinical use of metabolic drugs requires multifaceted evaluation.

**Table 3:** Intervention strategies targeting metabolic enzymes.

Targets of metabolism	Compounds	Pathways	References
Phosphoglucose-isomerase	2-DG	Glycolysis	(127)
PKM2	TT-232 Shikonin/alkannin	Glycolysis	(128, 129)
LDH	Oxamic acid Galloflavin	Glycolysis	(130, 131)
LDHA	FX11 Quinoline 3-sulfonamides	Glycolysis	(132, 133)
HK II	3-Bromopyruvate (3BP) Combination of 3-BrOP and rapamycin Combination of MGCD265 and erlotinib	Glycolysis	(134–137)
GLUT1	Phloretin Quercetin STF31 WZB117 Oxime-based GLUT1 inhibitors	Glycolysis	(138–142)
ACC	TOFA	Fatty acid metabolism	(143, 144)
Succinate dehydrogenase	Sodium malonate	TCA cycle	(145)
Fumarase	Fumaric acid esters	TCA cycle	(146)
Aconitase	Fluoroacetate	TCA cycle	(147)
FASN	C75	Fatty acid metabolism	(148)
SREBP	AM580	Fatty acid metabolism	(149)
HMG-CoA	Statins	Cholesterol metabolism	(150)
Glutaminase	BPTES	Amino acid metabolism	(108)

Ongoing antiviral research will focus on the metabolic enzymes involved in abnormal processes, such as glycolysis (34, 37, 63). Although virus infections induce aerobic glycolysis, several viruses utilize cellular mitochondrial function for replication. Therefore, targeting the glycolytic pathway is not the only therapeutic approach, and alternative antimetabolic approaches, such as targeting mitochondrial metabolism, including the pentose phosphate pathway, fatty acid synthesis, and amino acid metabolism, may be potential antiviral targets in the future. In summary, a more in-depth understanding of the regulatory mechanisms of metabolic alterations and viral replication will facilitate the development of antiviral drugs.

Currently, the precise mechanisms of metabolic reprogramming induced by viral infections remain largely unknown. In-depth exploration of these questions will enhance our understanding of virus-cell interactions, increase the possibility of future drug development targeting metabolism, and expand the library of drugs available for the treatment of viral infections.

## 7 Conclusions and perspectives

In this review, we have outlined the impact of cellular metabolism on viral infection and immunoevasion. The relationship between cell metabolism and viruses is very intricate. Viral infections can induce the changes in cellular metabolic pathways (**Table 4**), thereby providing the essential nutrients and energy for viral replication. When discussing the mechanisms of viral immunoevasion, we focus on key intermediate metabolic substances involved in several metabolic pathways hijacked by viruses, including glycolysis, the hexosamine pathway, the TCA cycle, the fatty acid synthesis pathway, the cholesterol metabolic pathway, the glutamine metabolic pathway, and the tryptophan metabolic pathway. These pathways not only provide nutrients and metabolites for the viral replication, but also participate in the regulation of immune responses.

**Table 4:** Cellular metabolism changes in viral infections.

Viruses	Up-regulated	Down-regulated	References
HIV-1	GLUT1, glutamine and PPAR-*γ*		(36, 37, 87, 107)
DENV	GLUT1 and HK2		(39)
RV	GLUT1 and glutamine		(17)
HBV	Lactate and HK2		(43, 44)
WSSV	GLUT1		(40)
EBV	GLUT1		(41)
HPV	HIF-1*α*		(65–67)
ARV	Glutamine and IDH3	LDHA	(48, 125)
IAV	Lactate, OGT, HK2, ACC and PPAR-*γ*	ApoE	(45, 50, 71, 81, 89, 93)
VSV	OGT and HIF-1A		(52, 53, 58)
KSHV	HIF-1*α*		(18)
SARA-CoV-2	Succinate, HIF-1*α* and IDO1		(59, 60, 62, 72, 119)
CSFV		LDHB	(46, 47)
ASFV	Lactate and glutamine		(27)
HCMV	PPAR-*γ* and glutamine		(88, 109)
HCV	ApoE		(90–92)
HSV-1	Succinate and glutamine		(72, 112)
ZIKV	Succinate		(72)
VACV	Succinate and glutamine		(39, 72, 106)
NDV	HIF-1*α*, SLC1A3 and MTHFD2		(58, 113, 123)
RGNNV	Glutamine		(108)
EV-71	Glutamine		(110)

Viruses cause reprogramming of glucose metabolism, which affects their immune evasion ability (151). More specifically, SARS-CoV-2 reprograms glucose metabolism and glycolysis can be hijacked by various viruses for favoring the viral infections (36, 37, 44, 45, 56, 152). The reprogramming promotes metabolic pathways and stabilizes genes involved in glucose transport and glycolysis, such as GLUT, LDH-A, and LDHB. HIF-1*α* activates the expression of a variety of glycolytic enzymes, including GLUT1, GLUT3, HK1, HK2, GAPDH, PGK1, PKM2, LDHA, and PDK1 (153–156). Importantly, HIF-1*α* not only increases glucose uptake and lactate production, but also blocks the TCA cycle and oxidative phosphorylation in mitochondria (156). Moreover, glycolytic flux and TCA cycle activity increase significantly with glucose uptake and lactate release (58). LDHA can enhance IFN-*γ* expression by upregulating histone acetylation (49, 157). Aerobic glycolysis can also promote IFN-*γ* production by binding GAPDH to the 3’UTR of IFN-*γ* mRNA (158). In addition, many intermediate metabolites, such as succinate, fumarate, itaconate, and *α*-ketoglutarate, are involved in immune activation or regulation. UDP-GlcNAc, the end product of the HBP, plays an important role in the process of viral inflammation through OGT (159). OGT mediates the transfer of UDP-GlcNAc to serine or threonine residues of target proteins to modify immune-related proteins (160) and coordinates glucose and glutamine metabolism induced by growth factors (32).

Viruses can utilize lipid-related factors for immunoevasion. For example, the deletion of *ACC* and *SCD2* can indirectly activate the cGAS-STING signaling pathway and enhance the host’s antiviral activities (81). Moreover, some viruses can antagonize the NF-κB or IFN-β signaling through regulating the lipid metabolism (87, 100). Amino acids are the key nutrients that can be converted into α-ketoglutaric acid to participate in the TCA cycle (108). Amino acid metabolism also plays an important role in the immunoevasion of viruses. Previous studies have shown that viruses can hijack glutamine and SLC1A3 (the amino acid transporter) to facilitate the viral latency and infection (112, 113).

In summary, there is a potential role for metabolism-related molecules in the development of antiviral drugs and diagnostic reagents by inducing host metabolic reprogramming to regulate viral replication (161). However, the application of immunotherapy in the treatment of viruses has not yet been extensively developed. Therefore, targeting immune cell function through metabolic modulation is a promising avenue for immunotherapy in the future (9, 72, 162).

## Author contributions

JL wrote the manuscript. YW, HD, SL and H-JQ revised this manuscript. All authors contributed to the article and approved the submitted version.
